# Dataset for unrevealing the application of multi-trait genotype-ideotype distance index and multi-trait index based on factor analysis and ideotype-design models in the identification of high-yielding and stable barley genotypes

**DOI:** 10.1016/j.dib.2025.111383

**Published:** 2025-02-11

**Authors:** Alireza Pour-Aboughadareh, Omid Jadidi, Bita Jamshidi, Jan Bocianowski, Janetta Niemann

**Affiliations:** a Seed and Plant Improvement Institute, Agricultural Research, Education and Extension Organization (AREEO), Karaj 3183964653, Iran; b Department of Plant Breeding and Biotechnology, Science and Research Branch, Islamic Azad University, Tehran 14778-93855, Iran; c Department of Food Security and Public Health, Khabat Technical Institute, Erbil Polytechnic University, Erbil 44001, Iraq; d Department of Mathematical and Statistical Methods, Poznań University of Life Sciences, 60-637 Poznań, Poland; e Department of Genetics and Plant Breeding, Poznań University of Life Sciences, 60-637 Poznań, Poland

**Keywords:** Genotype-by-environment interaction, Grain yield, AMMI model, BLUP model, Stability parameters

## Abstract

Dissecting the genotype-by-environment interaction (GEI) effects in multi-environmental trials (METs) is a critical step in any breeding program before introducing new commercial varieties for cultivation in specific regions or across diverse environments. This dataset explores the application of two novel selection models: the multi-trait genotype-ideotype distance index (MGIDI) and the multi-trait index based on factor analysis and ideotype-design (FAI-BLUP). These models incorporate comprehensive stability parameters to identify high-yielding and stable barley genotypes across varying environmental conditions. In both models, the first three factors (FAs) with eigenvalues greater than 1 accounted for 92.3% of the total variation. The BLUP-based parameters, along with grain yield (GY) and the mean variance component (Ɵ), showed a positive selection deferential (SD) and correlated with the second factor (FA2). Notably, these models identified G3, G10, and G14 as the most stable genotypes. In conclusion, this dataset underscores the utility of comprehensive stability parameters and advanced selection models in identifying high-yielding, stable genotypes within the framework of METs.

Specifications Table

**Table:** 

Subject	Agricultural and Biological Science
Specific subject area	Agronomy and Crop Science
Type of data	Table and Figure. Analyzed data.
Data collection	Grain yield data for 20 barley genotypes were collected from five locations in Iran's warm climate zone – Darab, Ahvaz, Moghan, Zabol, and Gonbad – over two cropping seasons (2020-2022). Field trials at the test locations were conducted using a randomized complete block design with three replicates. Detailed descriptions of the growth conditions and experimental layouts are provided in [1]. Following data collection, 32 stability parameters, encompassing non-parametric, parametric, and models-based approaches, were computed and subsequently analysed.
Data source location	The Seed and Plant Improvement Institute (SPII), part of the Agricultural Research, Education and Extension Organization (AREEO), Karaj, Iran conducted the study. Experimental data were derived from multi-location trials performed as five locations within the warm regions of Iran: Ahvaz (31° 19′ 13″ N 48° 40′ 09″ E), Darab (28° 45′ 07″ N 54° 32′ 40″ E), Zabol (37° 15′ 00″ N 55° 10′ 02″ E), Moghan (39° 38′ 54″ N 47° 55′ 03″ E), and Gonbad (31° 01′ 43″ N 61° 30′ 04″ E).
Data accessibility	Repository name: Dataset Data identification number: https://doi.org/10.17632/sv5kv6tk94.2 Direct URL to data: https://data.mendeley.com/datasets/sv5kv6tk94/2
Related research article	[1] S. Rahmati, R. Azizi-Nezhad, A. Pour-Aboughadareh, A. Etminan, L. Shooshtari. Analysis of genotype-by-environment interaction effect in barley genotypes using AMMI and GGE biplot methods, Heliyon, 10(2024) 38131. https://doi.org/10.1016/j.heliyon.2024.e38131

## 1 Value of the Data

• The dataset analyzed in this report indicates the importance dissection of the GEI effect in MET.
• This dataset reveals a breadth of type of stability parameters in selection of high-yielding and stable genotypes in MET.
• This dataset highlights the efficiency of selection models such as MGIDI and FAI-BLUP in crop breeding programs.
• As a remark outcome, integrating different stability parameters in a unique selection model can enhance the accuracy of selecting superior genotypes.

## 2 Background

Genotype-by-environment interaction (GEI) effects play a critical role in plant breeding programs, as they significantly influence the phenotypic expression of genotypes across various environments [2]. These interactions directly affect the selection and stability of desirable traits. Consequently, understanding GEI enables breeders to identify high-yielding genotypes with broad adaptability and stability, optimize breeding strategies, and enhance the efficiency of selection processes by accounting for environmental variability. In this context, METs are a fundamental component of plant breeding programs. METs involve evaluating a set of genotypes across various environments, including variations in location, year, or their combination, with the primary objective of analyzing and interpreting GEI effects [3]. Over recent decades, several statistical methods and models have been proposed to analyze the GEI effects and identify stable genotypes in METs. These methods and models can be broadly-categorized into two groups: (i) univariate approaches and (ii) multivariate approaches [3]. Among these, the GGE biplot analysis has emerged as a valuable graphical tool, enabling breeders to effectively select high-yielding and stable genotypes [4]. However, a comprehensive evaluation of genotypic responses is essential to fully assess the effect of GEI and identify superior genotypes. In a previous study [1], we employed the GGE biplot method to investigate GEI effect and identify high-yielding and stable barley genotypes, focusing on their adaptability in the warm climate of Iran. This dataset indicates the potential of integrating various stability parameters into a unified selection model, allowing breeders to select superior genotypes without relying solely on GGE biplot analysis. Additionally, evaluating the efficiency of these selection models represents another key objective of this report.

## 3 Data Description

The dataset presented in this article comprises four tables and one figure. Table 1 indicates the results of the combined analysis of variance (ANOVA), which reveals significant effects for environments (E), genotypes (G), and their interaction (GEI). These findings are further validated by the additive main effects and multiplicative interaction (AMMI) model. A detailed dissection of the GEI effect using the AMMI model demonstrates that the interaction can be partitioned into three significant interaction principal component axes (IPCAs). Specifically, IPCA1, IPCA2, and IPCA3 accounted for 31.83%, 24.80%, and 12.36% of the total GEI variation, respectively (Table 1). Table 2 presents the estimated grain yield (GY) values and 32 stability parameters for the genotypes evaluated across ten test environments.

**Table 1:** The results of combined analysis of variance (ANOVA) and AMMI model for grain yield in the investigated genotypes of barley across different regions in the warm climate of Iran.

	SOV	Df	SS	MS	*F*-value	Probability	TSS (%)
Combined ANOVA	Environment (E)	9	1033.8	114.86	273.14	**	
	Replication (R)/E	20	24.1	1.20	2.86	**	
	Genotype (G)	19	25.2	1.33	3.16	**	
	G × E Interaction	171	123.8	0.724	1.72	**	
	Residual	380	159.8	0.42			
AMMI model	Total	599	1366.7	2.28			
	Treatments	199	1182.9	5.94	14.13		
	G	19	25.2	1.33	3.16	**	
	E	9	1033.8	114.87	95.51	**	
	Block	20	24.1	1.2	2.86		
	G × E Interaction	171	123.8	0.72	1.72	**	10.47
	IPCA1	27	39.4	1.46	3.47	**	31.83
	IPCA2	25	30.7	1.23	2.92	**	24.80
	IPCA3	23	15.3	0.66	1.58	*	12.36
	Residuals	96	38.5	0.4	0.95		
	Error	380	159.8	0.42			

* and ** Significant at *P* < 0.01, respectively. SOV, df, SS, MS, and TTS indicate source of variation, the number of degree of freedom, sum of square, mean sum of square, and the total sum of square, respectively.

**Table 2:** Estimated values of various stability parameters for the investigated barley genotypes.

Code	GY	θ	HMGV	RPGV	HMRPGV	WAASB	S^(1)^	S^(2)^	S^(3)^	S^(6)^	NP(1)	NP^(2)^	NP^(3)^	NP^(4)^	$W^{2}$	$\sigma^{2}$	$S_{di}^{2}$
G1	4.66	0.24	4.07	1.02	1.02	0.18	4.73	17.43	14.14	2.99	3.20	0.43	0.40	0.43	1.81	0.21	0.26
G2	4.52	0.25	3.96	0.99	0.99	0.09	4.11	12.06	11.42	3.05	2.70	0.32	0.36	0.43	0.64	0.07	0.09
G3	4.95	0.25	4.24	1.06	1.06	0.20	4.51	15.88	9.59	2.48	5.30	0.37	0.39	0.30	1.39	0.16	0.17
G4	4.63	0.24	4.04	1.01	1.01	0.26	6.73	41.39	29.80	4.08	4.30	0.30	0.47	0.54	2.64	0.31	0.36
G5	4.66	0.24	4.07	1.02	1.01	0.17	7.27	40.94	29.48	4.64	5.50	0.35	0.49	0.58	1.68	0.19	0.24
G6	4.41	0.24	3.84	0.97	0.97	0.22	7.27	39.17	41.47	6.00	5.00	0.74	0.72	0.85	2.40	0.28	0.34
G7	4.53	0.21	3.89	0.99	0.99	0.37	9.38	64.18	58.94	7.14	7.10	0.64	0.78	0.96	6.54	0.79	0.93
G8	4.40	0.25	3.84	0.97	0.97	0.09	5.38	22.71	26.89	4.79	3.50	0.75	0.58	0.71	0.75	0.08	0.11
G9	4.61	0.23	4.10	1.01	1.01	0.27	8.53	52.04	41.09	5.33	6.40	0.62	0.65	0.75	3.74	0.45	0.51
G10	4.72	0.25	4.11	1.02	1.02	0.14	5.67	22.68	16.07	3.23	3.90	0.38	0.38	0.45	1.03	0.11	0.14
G11	4.50	0.25	4.00	1.00	1.00	0.11	6.69	33.17	28.43	4.76	5.20	0.46	0.59	0.64	1.17	0.13	0.13
G12	4.43	0.24	3.95	0.98	0.98	0.17	6.00	29.16	25.23	4.35	5.10	0.33	0.59	0.58	2.16	0.25	0.24
G13	4.56	0.24	3.99	1.00	1.00	0.16	5.53	24.28	20.81	3.71	3.70	0.28	0.49	0.53	1.52	0.17	0.19
G14	5.03	0.24	4.35	1.08	1.07	0.22	4.33	13.88	7.86	1.96	4.80	0.52	0.37	0.27	1.69	0.20	0.22
G15	4.48	0.24	3.91	0.98	0.98	0.20	7.18	37.83	40.06	5.76	5.30	0.60	0.72	0.84	2.46	0.29	0.33
G16	4.56	0.22	3.93	1.00	0.99	0.35	8.16	46.01	38.70	5.33	6.50	0.57	0.67	0.76	5.35	0.65	0.76
G17	4.75	0.25	4.09	1.03	1.03	0.10	6.53	30.27	21.62	3.81	5.30	0.36	0.46	0.52	1.39	0.16	0.19
G18	4.14	0.25	3.69	0.93	0.93	0.18	4.62	19.29	33.38	5.85	4.40	1.51	0.97	0.89	1.47	0.17	0.16
G19	4.22	0.25	3.77	0.95	0.95	0.08	2.93	7.38	11.86	3.43	3.30	1.04	0.72	0.52	0.41	0.04	0.03
G20	4.45	0.25	3.95	0.99	0.99	0.15	4.56	16.01	15.49	3.12	3.50	0.34	0.49	0.49	1.05	0.12	0.14


Code	CV	*θ'*	KR	ASTAB	ASI	ASV	AVAMGE	DA	DZ	EV	FA	MASI	MASV	SIPC	ZA	WAAS	
G1	30.55	0.23	18.00	0.54	0.11	0.43	2.89	1.18	0.47	0.07	1.38	0.13	0.80	1.08	0.12	0.30	
G2	30.84	0.16	14.00	0.08	0.06	0.25	1.14	0.49	0.17	0.01	0.24	0.06	0.50	0.43	0.05	0.14	
G3	31.81	0.21	10.00	0.35	0.13	0.52	2.48	1.01	0.35	0.04	1.01	0.14	0.79	1.00	0.13	0.33	
G4	34.49	0.28	24.00	0.52	0.18	0.72	3.12	1.28	0.42	0.06	1.63	0.19	1.07	1.24	0.17	0.43	
G5	32.26	0.23	17.00	0.21	0.11	0.44	1.92	0.80	0.27	0.02	0.65	0.12	0.59	0.76	0.10	0.25	
G6	34.81	0.27	32.00	0.34	0.16	0.64	2.80	1.06	0.32	0.03	1.13	0.16	1.05	0.89	0.13	0.34	
G7	35.30	0.51	31.00	1.88	0.40	1.60	7.02	2.53	0.75	0.19	6.42	0.40	1.93	2.18	0.31	0.82	
G8	32.62	0.17	21.00	0.01	0.03	0.13	0.50	0.19	0.05	0.00	0.04	0.03	0.14	0.13	0.02	0.06	
G9	30.11	0.35	26.00	1.15	0.22	0.89	4.40	1.82	0.64	0.14	3.32	0.23	1.78	1.74	0.21	0.54	
G10	31.96	0.19	8.00	0.13	0.11	0.43	1.75	0.66	0.19	0.01	0.44	0.11	0.55	0.50	0.08	0.21	
G11	28.09	0.20	19.00	0.04	0.03	0.14	0.85	0.32	0.12	0.00	0.10	0.04	0.22	0.30	0.04	0.09	
G12	27.90	0.25	30.00	0.45	0.16	0.66	3.11	1.19	0.38	0.05	1.42	0.16	1.33	0.78	0.10	0.26	
G13	34.40	0.22	19.00	0.28	0.17	0.68	2.84	1.01	0.28	0.03	1.02	0.17	0.69	0.66	0.10	0.28	
G14	31.44	0.23	13.00	0.46	0.18	0.71	3.37	1.21	0.39	0.05	1.46	0.18	0.84	1.07	0.14	0.37	
G15	35.60	0.27	30.00	0.63	0.19	0.78	3.90	1.41	0.45	0.07	1.99	0.20	1.54	1.09	0.15	0.37	
G16	35.99	0.44	29.00	1.67	0.30	1.22	5.96	2.25	0.76	0.19	5.07	0.32	1.94	2.24	0.29	0.75	
G17	31.67	0.21	10.00	0.01	0.02	0.09	0.42	0.16	0.06	0.00	0.03	0.02	0.12	0.16	0.02	0.05	
G18	29.90	0.21	29.00	0.12	0.09	0.38	1.46	0.62	0.19	0.01	0.38	0.09	0.48	0.56	0.08	0.20	
G19	29.66	0.15	20.00	0.02	0.03	0.12	0.71	0.26	0.10	0.00	0.07	0.03	0.17	0.22	0.03	0.07	
G20	29.82	0.19	20.00	0.19	0.13	0.53	2.09	0.82	0.23	0.02	0.67	0.13	0.61	0.65	0.10	0.26	

See Table 4 for abbreviations

**Fig. 1:**
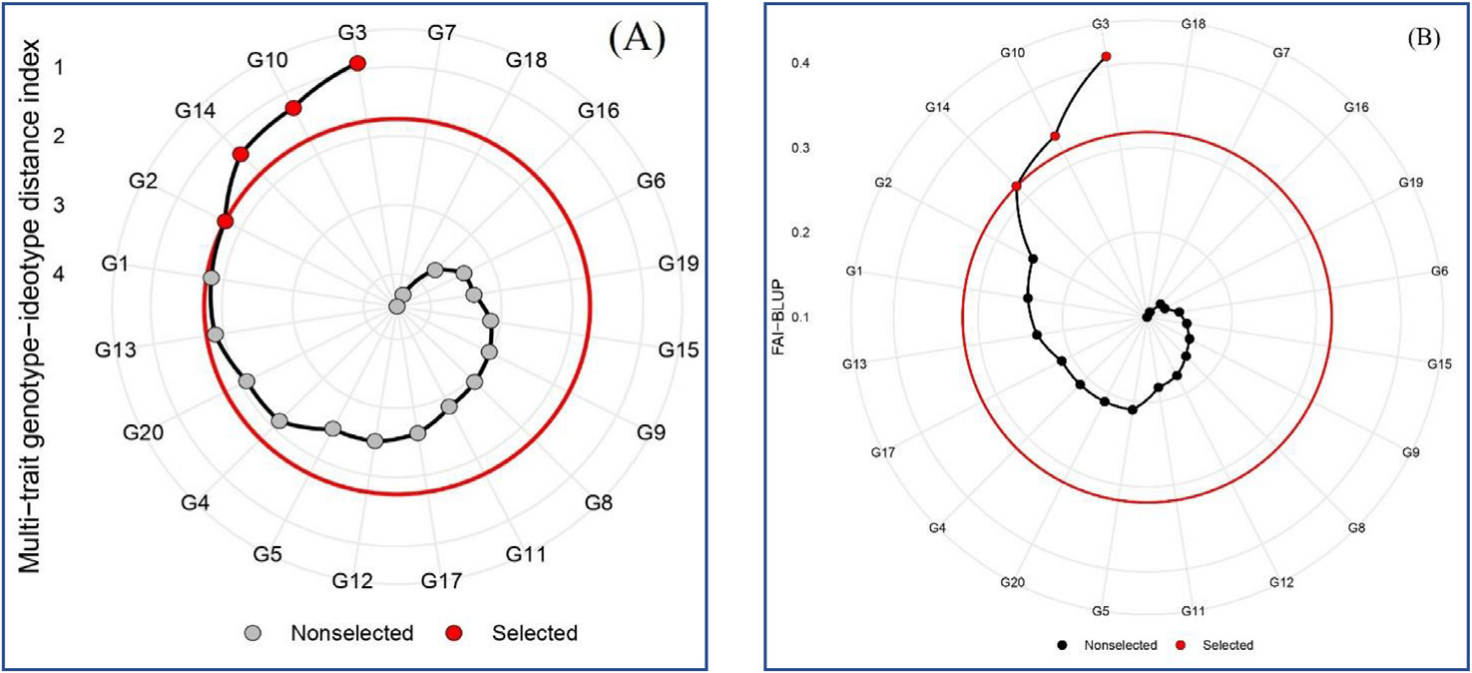
Identified high-yielding and stable barley genotypes based on the ranking patterns obtained by the MGIDI (A) and FAI-BLUP (B) models. The selected genotypes (G2, G3, G10, and G14) have been highlighted in red. The central red circle is a cut of line based on the SI = 20%.

The results indicated that certain stability parameters exhibited similar ranking patterns across the tested genotypes. For example, genotypes G14, G3, G17, and G10, which had the highest GY, were identified as the most stable genotypes based on the HMGV, RPGV, and HMRPGV parameters. Similarly, the top-ranked genotypes based on the S^(1)^, S^(2)^, and S^(3)^ stability parameters were G2, G3, G14, and G19. Stability parameters such as Wricke's ecovalence (W^2^), deviation from regression (S_d_^2^), Shukla's stability variance (σ^2^), and the mean variance component (Ɵ) showed similar results in identifying stable genotypes. Additionally, all AMMI-based stability parameters collectively identified G8, G11, G17, and G19 as the most stable genotypes. Given the different outcomes of various stability parameters in identifying stable genotypes, the MGIDI and FAI-BLUP models were utilized to integrate all stability parameters and select ideal genotypes. Table 3 presents the factor loadings and eigenvalues for the main effective factors in the MGIDI and FAI-BLUP models. In both models, the first three factors (FAs) with eigenvalues greater than 1 accounted for 92.3% of the total variation. Notably, BLUP-based parameters, along with GY and the mean variance component (Ɵ), demonstrated a positive selection deferential (SD) and were associated with the second factor (FA2). Fig. 1 illustrates the screening results for the barley genotypes based on the MGIDI (A) and FAI-BLUP (B) models. The central red circles represent the cutoff thresholds determined by the selection pressures (in the present study, SI was determined as 20%). Both models consistently identified G3, G10, G14, and G2 as the most stable genotypes. These findings align with a previous study by Rahmati et al. [1], which also identified G3, G10, and G14 as high-yielding genotypes with excellent adaptability to diverse environments within Iran's warm climate. This consistency further supports the reliability of the results presented in this dataset.

**Table 3:** Factor loadings and sense patterns, and selection deferential (SD) for stability parameters in the MGIDI and FAI-BLUP models.

Parameter	Sense	MGIDI model				FAI-BLUP model			
		Factor	Xo	Xs	SD	Factor	Xo	Xs	SD
*θ*	Increase	FA1	0.24	0.25	0.01	FA1	0.24	0.25	0.01
WAASB	Decrease	FA1	0.19	0.16	-0.02	FA1	0.19	0.16	-0.02
$W^{2}$	Decrease	FA1	2.06	1.19	-0.88	FA1	2.06	1.19	-0.88
$\sigma^{2}$	Decrease	FA1	0.24	0.14	-0.11	FA1	0.24	0.14	-0.11
$S_{di}^{2}$	Decrease	FA1	0.28	0.16	-0.12	FA1	0.28	0.16	-0.12
CV	Decrease	FA1	32.00	31.50	-0.45	FA1	31.96	31.51	-0.45
*θ'*	Decrease	FA1	0.25	0.20	-0.05	FA1	0.25	0.20	-0.05
ASTAB	Decrease	FA1	0.45	0.26	-0.20	FA1	0.45	0.26	-0.20
ASI	Decrease	FA1	0.14	0.12	-0.02	FA1	0.14	0.12	-0.02
ASV	Decrease	FA1	0.57	0.48	-0.09	FA1	0.57	0.48	-0.09
AVAMGE	Decrease	FA1	2.64	2.18	-0.45	FA1	2.64	2.19	-0.45
DA	Decrease	FA1	1.01	0.84	-0.17	FA1	1.01	0.84	-0.17
DZ	Decrease	FA1	0.33	0.28	-0.05	FA1	0.33	0.28	-0.05
EV	Decrease	FA1	0.05	0.03	-0.02	FA1	0.05	0.03	-0.02
FA	Decrease	FA1	1.42	0.79	-0.64	FA1	1.42	0.79	-0.64
MASI	Decrease	FA1	0.15	0.12	-0.02	FA1	0.15	0.12	-0.02
MASV	Decrease	FA1	0.86	0.67	-0.19	FA1	0.86	0.67	-0.19
SIPC	Decrease	FA1	0.88	0.75	-0.13	FA1	0.88	0.75	-0.13
ZA	Decrease	FA1	0.12	0.10	-0.02	FA1	0.12	0.10	-0.02
WAAS	Decrease	FA1	0.31	0.26	-0.04	FA1	0.31	0.26	-0.04
YS	Increase	FA2	4.56	4.80	0.24	FA2	4.56	4.81	0.24
HMGV	Increase	FA2	3.99	4.16	0.18	FA2	3.99	4.17	0.18
RPGV	Increase	FA2	1.00	1.04	0.04	FA2	1.00	1.04	0.04
HMRPGV	Increase	FA2	1.00	1.04	0.04	FA2	1.00	1.04	0.04
NP^(2)^	Decrease	FA2	0.55	0.40	-0.15	FA2	0.55	0.40	-0.15
NP^(3)^	Decrease	FA2	0.56	0.38	-0.19	FA2	0.56	0.38	-0.19
NP^(4)^	Decrease	FA2	0.60	0.36	-0.24	FA2	0.60	0.36	-0.24
KR	Decrease	FA2	21.00	11.20	-9.75	FA2	21.00	11.25	-9.75
S^(1)^	Decrease	FA2	6.01	4.66	-1.35	FA2	6.01	4.66	-1.35
S^(2)^	Decrease	FA3	29.30	16.10	-13.20	FA2	29.29	16.13	-13.16
S^(3)^	Decrease	FA3	26.10	11.20	-14.90	FA2	26.12	11.24	-14.88
S^(6)^	Decrease	FA3	4.29	2.68	-1.61	FA2	4.29	2.68	-1.61
NP^(1)^	Decrease	FA3	4.70	4.18	-0.53	FA2	4.70	4.18	-0.53

See Table 4 for abbreviations

## 4 Experimental Design, Materials and Methods

### 4.1 Descriptions of test genotypes and experimental sites

The plant materials used in this study consisted of eighteen promising barley genotypes, along with two local varieties [Oxin and Golchin] which served as reference genotypes. Detailed information on the pedigree of the tested genotypes is provided in Supplementary Table S1. The reference genotypes, recently released varieties, are characterized by high performance, stability, and broad adaptability across the warm regions of Iran. Field experiments were conducted at five locations within Iran's warm climate, which included Zabol, Ahvaza, Darab, Moghan, and Gonbad, over two cropping seasons (2022-2022 and 2022-2023). Geographical information for the test environments is presented in Table S1. A randomized complete block with three replications was employed as each location. The seed density was standardized at 350 seeds per square meter (150 kg ha^−1^) across all experiments. Each experimental plot consisted of six rows, each 5 meters in length, with 20 cm spacing between rows. The same seed source was used uniformly across all trials. Planting and harvesting processes were performed using a small-scale experimental planter and combine harvester (Wintersteiger, Ried, Austria). Finally, grain yield (GY) for each genotype was calculated in tons per hectare. More information regarding culturing practices and other information is available in Rahmati et al. [1].

### 4.2 Stability parameters

After collecting the experimental data, a combined analysis of variance (ANOVA) was conducted to assess the main effects of environments (E), genotypes (G), and their interaction (GEI) on the grain yield data. Additionally, an AMMI analysis of variance was performed to extract the significant principal component interactions (IPCAs). Subsequently, several parametric and non-parametric stability parameters were estimated, as presented in Table 4. Details descriptions of each stability parameter and its corresponding mathematical formula can be found in Pour-Aboughadareh et al. (2020). All stability parameters were calculated using the STABILITYSOFT software [2] and the ‘metan’ package [5] in R software [6]. Finally, the selection models MGIDI and FAI-BLUP were estimated as described by Olivoto et al. [7] and Rocha et al. [8] using the ‘metan’ packages in R.

**Table 4:** List of parametric and non-parametric stability statistics to analyze GEI effect in this dataset.

Satbility parameter	Symbol	Pattern of selection	Reference
Mean variance component	Θ	Minimum value	[9]
GE variance component	θ'	Maximum value	[10]
Wricke's ecovalence	$W^{2}$	Minimum value	[11]
Deviation from regression	$S_{di}^{2}$	Minimum value	[12]
Shukla's stability variance	$\sigma^{2}$	Minimum value	[13]
Coefficient of variance	CV	Minimum value	[14]
Nassar and Huhn's and Huhn's statistics	S^(1, 2, 3, 6)^	Minimum value	[15,16]
Kang's rank-sum	KR	Minimum value	[17]
Yield stability index	YS	Maximum value	[18]
Averages of the squared eigenvector values	Ev	Minimum value	[19]
Thennarasu's non-parametric statistics	NP^(1-4)^	Minimum value	[20]
Sums of the absolute value of the IPC scores	SIPC	Minimum value	[21]
Sum across environments of the GEI modelled by AMMI	AMGE	Minimum value	[21]
Distance of IPCAs point with origin in space	D	Minimum value	[22,23]
AMMI stability value	ASV	Minimum value	[24]
AMMI Based Stability Parameter	ASTAB	Minimum value	[25]
Harmonic mean of genotypic values	HMGV	Maximum value	[26]
Relative performance of genotypic values	RPGV	Maximum value	[26]
Harmonic mean of RPGV	HMRPGV	Maximum value	[26]
Genotype stability index	GSI	Maximum value	[27]
Modified AMMI Stability Value	MASV	Minimum value	[28]
Absolute value of relative contribution of IPCAs	ZA	Minimum value	[28]
Sum across environments of absolute value of GEI modelled by AMMI	AVAMGE	Minimum value	[28]
AMMI stability index	ASI	Minimum value	[29]
Modified AMMI stability index	MASI	Minimum value	[30]
Weighted average of absolute scores	WAAS	Minimum value	[31]
Weighted average absolute scores of BLUP	WAASB	Minimum value	[31]

## Limitations

Not applicable.

## Ethics Statement

All authors have read and follow the ethical requirements for publication in Data in Brief and our work meets these requirements. Our work does not involve studies with animals and humans.

## CRediT authorship contribution statement

**Alireza Pour-Aboughadareh:** Conceptualization, Methodology, Software, Investigation, Data curation, Writing – review & editing. **Omid Jadidi:** Methodology, Software. **Bita Jamshidi:** Software, Writing – original draft. **Jan Bocianowski:** Validation, Writing – review & editing. **Janetta Niemann:** Validation, Writing – review & editing.

## Data Availability

Dataset for unrevealing the application of MGIDI and FAI-BLUP models in the identification of high-yielding and stable barley genotypes (Original data).

## Appendix Supplementary Materials

Image, application 1
