# Supplementary material for: Dataset for unrevealing the application of multi-trait genotype-ideotype distance index and multi-trait index based on factor analysis and ideotype-design models in the identification of high-yielding and stable barley genotypes
Source: Data Brief. 2025 Feb 11;59:111383. doi: 10.1016/j.dib.2025.111383 (PMC11919321; doi:10.1016/j.dib.2025.111383)
Supplement: Supplementary file 1 [file mmc1.docx]

| **Table S1**  The list of studied barley genotypes and test environments. | | | | | |
| --- | --- | --- | --- | --- | --- |
| Code | Pedigree | | | | |
| G1 | Oxin [Reference] | | | | |
| G2 | Golchin [Reference] | | | | |
| G3 | Rojo/3/LB.IRAN/Una8271//Gloria"S"/Com"S"/4/ Briges | | | | |
| G4 | Rojo/3/LB.IRAN/Una8271//Gloria"S"/Com"S"/4/Rihane-03 | | | | |
| G5 | Zarjau/80-5151//OK84817ICBH94-0402-0AP-0AP-17AP-0AP-12AP-11AP-0AP-0TR-0TR-0AREC | | | | |
| G6 | Zarjau/80-5151//OK84817ICBH94-0402-0AP-0AP-17AP-0AP-12AP-16AP-0AP-0TR-0TR-0AREC | | | | |
| G7 | Lignee 527/NK1272//JLB 70-63/3/Rhn-03//Lignee527/As45 | | | | |
| G8 | KAROON/KAVIR//Rhodes'S'//Tb/Chzo/3/Gloria'S' /4/Sahra/5/Yousef | | | | |
| G9 | Anoidium/Arbayan-01/3/Lignee527/NK1272//JLB70-63/4/ Beecher | | | | |
| G10 | Anoidium/Arbayan-01/3/Lignee527/NK1272//JLB70-63/4/Bgs/Dujia//L.1242 | | | | |
| G11 | Bgs/Dajia//L.1242/3/(L.B.IRAN/Una8271//Gloria'S'/3/Alm/Una80//....)/4/Nosrat/5/Rhn-03//L.527/NK1272 | | | | |
| G12 | CIRU/TOCTE | | | | |
| G13 | Courlis/Rhn-03//Jonoob | | | | |
| G14 | Zahak/4/Bgs/Dajia//L.1242/3/L.B.IRAN/Una8271//Gloria'S'/3/Alm/Una80//....)/4/Rojo… | | | | |
| G15 | Rojo/3/LB.IRAN/Una8271//Gloria"S"/Com"S"/4/Gloria'S'/Copal'S'//As46/Aths/3/Rhn-03 | | | | |
| G16 | Rojo/3/LB.IRAN/Una8271//Gloria"S"/Com"S"/4/Anoidium/Arbayan-01/3/Lignee527/… | | | | |
| G17 | Merzaga(Orge077)/Alanda-01 ICB98-0908-0AP-13AP-0AP-3TR-10AP-0AP-0TR-0TR | | | | |
| G18 | PENCO/CHEVRON-BAR/6/P.STO/3/LBIRAN/UNA80//LIGNEE640/4/BLLU/5/PETUNIA 1 | | | | |
| G19 | (Salt-4)LB.Iran/Una 8271//Gloria"S"/Come"s"-11M/3/Kavir/4/Karoon | | | | |
| G20 | W-98-10 | | | | |
|  | **Environments** | | | | |
| No. | Location | Year | Coordinates | Geographical position | Climate condition |
| 1 | Ahvaz | 2021-2022 | 31° 19' 13'' N 48° 40' 09'' E | South | Warm and dry |
| 2 |  | 2022-2023 |  |  |  |
| 3 | Darab | 2021-2022 | 28° 45' 07'' N 54° 32' 40'' E | South | Warm and dry |
| 4 |  | 2022-2023 |  |  |  |
| 5 | Gonbad | 2021-2022 | 31° 01' 43'' N 61° 30' 04'' E | Northeast | Warm and humid |
| 6 |  | 2022-2023 |  |  |  |
| 7 | Zabol | 2021-2022 | 37° 15' 00'' N 55° 10' 02'' E | Southeast | Warm and dry |
| 8 |  | 2022-2023 |  |  |  |
| 9 | Moghan | 2021-2022 | 39° 38' 54'' N 47° 55' 03'' E | Northwest | Warm and humid |
| 10 |  | 2022-2023 |  |  |  |
